# Molecular characterisation and liquid biomarkers in Carcinoma of Unknown Primary (CUP): taking the ‘U’ out of ‘CUP’

**DOI:** 10.1038/s41416-018-0332-2

**Published:** 2018-12-23

**Authors:** Alicia-Marie Conway, Claire Mitchell, Elaine Kilgour, Gerard Brady, Caroline Dive, Natalie Cook

**Affiliations:** 1 The Christie NHS Foundation Trust, Wilmslow Road, Manchester, M20 4BX UK; 2 The University of Manchester, Oxford Road, Manchester, UK; 3 Cancer Research UK Manchester Institute, Alderley Park, Alderley Edge, Macclesfield, Cheshire, SK10 4TG UK

**Keywords:** Cancer of unknown primary, Tumour biomarkers, Cancer genomics

## Abstract

Cancers of Unknown Primary (CUP) comprise a heterogeneous clinical entity of confirmed metastatic cancer where the primary site of origin is undetectable. It has a poor prognosis with limited treatment options. CUP is historically under-researched; however, understanding its biology has the potential to not only improve treatment and survival by implementation of biomarkers for patient management, but also to greatly contribute to our understanding of carcinogenesis and metastasis across all cancer types. Here we review the current advances in CUP research and explore the debated hypotheses underlying its biology. The evolution of molecular profiling and tissue-of-origin classifiers have the potential to transform the diagnosis, classification and therapeutic management of patients with CUP but robust evidence to support widespread use is lacking. Precision medicine has transformed treatment strategy in known tumour types; in CUP, however, there remains a clinical need for a better understanding of molecular characteristics to establish the potential role of novel or existing therapeutics. The emergence of liquid biopsies as a source of predictive and prognostic biomarkers within known tumour types is gaining rapid ground and this review explores the potential utility of liquid biopsies in CUP.

## Introduction

Cancer of Unknown Primary (CUP) is a rare heterogeneous clinical syndrome of metastatic cancers for which the primary site of origin is elusive. It is an under-researched entity, where little is understood of its biology and debate remains as to its true definition and classification. Two predominant theories exist: that CUP tumours are metastatic tumours that have arisen from a small undetectable or regressed primary lesion; or that CUP is a single metastatic entity, with no primary tumour that exists. If the metastatic tumours originated from a primary tumour, be it regressed or dormant, then CUP tumours may be more biologically similar or molecularly traceable to the primary, and therefore may respond to treatments aimed at the primary. This makes CUP a syndrome of distinct tumour types that require site-specific therapy. However, if CUP is a single cancer entity it is hypothesised that a shared molecular signature may exist to explain the aggressive phenotype. This view suggests treating tumours based on primary tumour type will not improve outcomes.

Current treatments for CUP depend on individual clinical presentation; however, most patients have poor prognosis disease with no consensus of ‘optimal’ chemotherapy, lack of robust prognostic and predictive biomarkers, and no access to targeted or immune-therapies. Research within CUP is hampered by the heterogeneity of the condition and there have been few advancements in treatments when compared to other metastatic cancers such as lung. In addition to the recent review of current advances in molecular characterisation and tissue-of-origin (TOO) research within CUP^1^, we will explore how novel trial design and personalised medicine could improve treatment strategies for patients with CUP. We will highlight the rapidly evolving field of liquid biopsies for diagnosis and monitoring and the potential applications for them in CUP.

## CUP diagnosis and management

CUP make up 3–5% of total cancer diagnoses worldwide.^2^ Incidence has been decreasing since the early 1990s, which likely reflects improvements in primary cancer site determination. Despite this improvement CUP remains the fifth leading cause of cancer death in the UK, carrying a poor prognosis with a median survival of 6-16 months.^2,3^ Patients with CUP have disseminated cancer at presentation where the originating site of the tumour is not readily detectable; this presentation is initially defined as malignancy of unknown origin (MUO). In the UK, MUO represents ~15% of all new cancer diagnoses.^4^ Patients with MUO undergo appropriate radiological investigations, tumour marker assessment and biopsy, determined by clinical presentation, in search of the cancer’s TOO. For the majority (two thirds) of patients presenting with MUO, these investigations will reveal or be suggestive of the tumour’s primary TOO. For the remaining patients a diagnosis of confirmed CUP is made.^5^

**Fig. 1:**
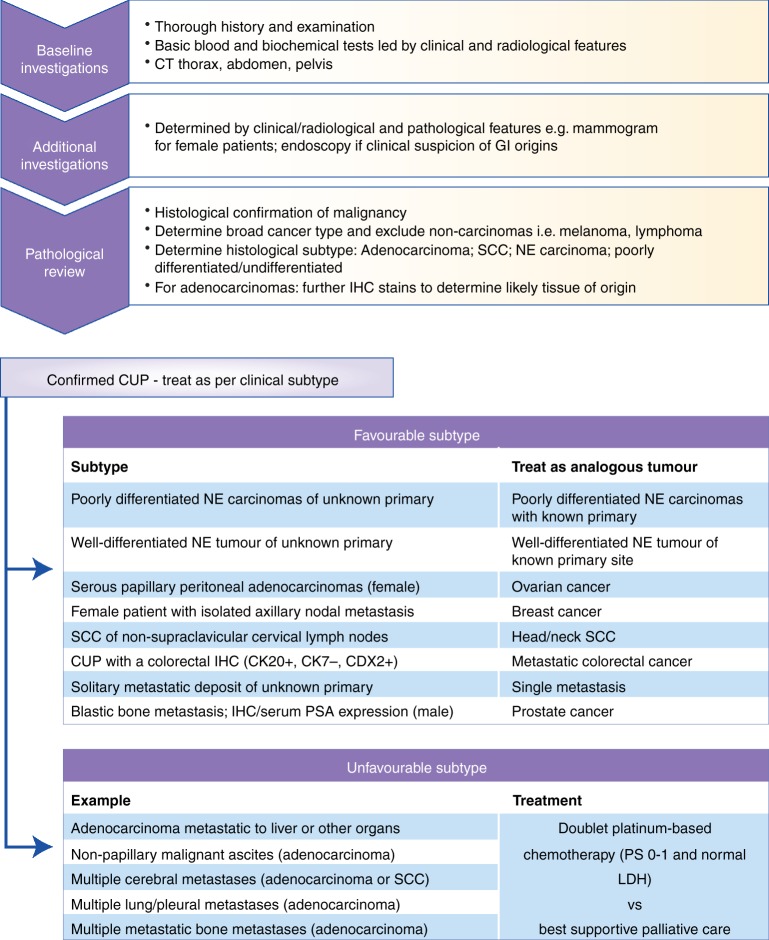
Current management and treatment options for favourable and unfavourable subtypes of CUP. Following tissue biopsy diagnosis of confirmed CUP, patients with favourable clinical features are treated as the analogous tumour type. Those patients within the unfavourable clinical subtype can be offered palliative chemotherapy if they have good performance status. There is no standard second line therapy. IHC immunohistochemistry, PS performance status, CK cytokeratin, LDH lactate dehydrogenase, SCC squamous cell carcinoma

The pathological review of tumour tissue is key to determining the TOO; however, given there is no international consensus describing the pathological approach, combined with technical variability and subjective interpretation of immunohistochemistry (IHC), diagnosis is challenging.^5^ Current European and US guidelines recommend a meticulous, step-wise histopathological approach including assessing tissue morphology and appropriate application of immunohistochemistry (IHC) to not only confirm malignancy, but exclude highly treatable non-carcinomas (i.e. lymphoma, sarcoma and melanoma) (reviewed in^5^). By definition CUP tumours are therefore carcinomas of unknown primary, with other cancer types excluded (Fig. 1). The most common histological subtypes of confirmed CUP are adenocarcinomas (60%), squamous (5%) and neuroendocrine (5%) cancers, with the remaining 30% comprising of poorly/undifferentiated carcinomas. For confirmed adenocarcinoma, additional appropriate IHC markers may determine the likely TOO. These include cytokeratin-7 and 20 (CK7/CK20) followed by further tissue-specific markers; for example, TTF1, ER and PR, and PSA, which can identify lung, breast and gynaecological, and prostate cancers, respectively.^5^

Patients with confirmed CUP are sub-classified into two subgroups based on histopathological and clinical features: favourable and unfavourable (see Fig. 1). Patients with a favourable subtype (~20%) have low volume, predominantly nodal disease with a pattern of metastatic spread that is similar to a known tumour type. Patients are treated in line with this analogous tumour type and subsequently exhibit survival similar to that tumour type.^6^ Patients with unfavourable CUP subtype (~80%) are more likely to have visceral disease, high tumour burden and exhibit poor response to standard chemotherapy. Prognosis is much shorter (6-12 months) than the favourable subtype and, for patients who do not undergo treatment, life expectancy can be as little as 4 weeks.^7^ Traditionally, patients within the unfavourable group have been treated as a single cancer entity due to shared clinical features, namely a short clinical history, atypical metastatic distribution, aggressive disease and an undetectable primary tumour. Patients with a good performance status and normal Lactate Dehydrogenase (LDH) can be considered for palliative chemotherapy. There is a lack of international consensus guiding optimal chemotherapy selection, but standard treatment for the unfavourable subtype is currently doublet chemotherapy, usually containing platinum and/or taxane, with response rates of 25-40% and median overall survival of 9 months. Multiple phase II studies have found no superior combination cytotoxics, and there is no standard second line treatment.^8^

## The biology of CUP

Little is understood of the disease pathogenesis and the biology explaining the CUP phenotype is debated. Two predominant hypotheses exist: 1) CUP tumours arise from a small, dormant or regressed primary tumour that is undetectable and are therefore a group of distinct primaries with an undetectable primary lesion. 2) No primary tumour exists or will exist, and ‘true’ CUP tumours arise independently to a tumour mass and are biologically distinct from other metastatic tumours.

The biology underlying early dissemination from a small/regressed primary lesion is not easily explained and research is hampered by the paradox of a lack of primary tissue. Interestingly, in around one quarter of patients with CUP, the primary tumour lesion becomes clinically or radiologically detectable later in the patient’s lifetime. Furthermore, autopsy studies have identified a primary tumour lesion in 50-80% of patients with previously diagnosed CUP; these are predominantly small primary lesions of the pancreas, lung or colon.^9–11^ It is notable, however, that despite improvements in imaging and histopathological diagnosis, up to 50% of patients with CUP do not have a primary tumour detected at autopsy. Additionally, in cases of occult breast cancer, which represent 0.3–1% of all breast cancer cases, frequently no primary breast lesion is found at diagnostic imaging or pathological review of mastectomy specimens.^12^

Theories of cancer dissemination are debated around known tumour types. The traditional view of metastatic progression is a stepwise process of progressive molecular aberrations initiating carcinogenesis, progressing to autonomous proliferation and invasion with subsequent tumour cell spread at a late stage of tumour growth (termed linear progression). Subsequent metastases are molecularly similar to their primary tumour and metastatic potential appears to be linked to primary tumour size. However, this view has been challenged by pre-clinical and clinical data in many types of cancers including melanoma, breast, pancreatic and small cell lung cancer.^13–16^

Parallel progression describes dissemination of primary tumour cells as an early event where subsequent clonal evolution of the metastasis is distinctly different to that of the primary tumour.^17^ Breast cancer tumours are felt to have early metastatic potential and disseminated cancer disseminated tumour cells (DTCs) are found in the bone marrow of patients with localised breast cancer; often remaining dormant for many years.^15,18,19^ Mouse models of melanoma have also demonstrated cancer cell dissemination as an early step in primary tumour development, with DTC dormancy being maintained by immune cells during immunosurveillance.^20^ In other cancer types, dissemination is early and rapid. In mouse models of pancreatic cancer, tumour cells disseminate early and metastatic tumour formation can precede the formation of the primary pancreatic tumour.^16^ Parallel progression better describes how a small, undetectable primary tumour could lead to aggressive and widespread metastasis, such as that found in patients with CUP.

Spontaneous tumour regression has been reported in several tumour types but is best described in cases of melanoma, where 5% of metastatic cases present with an occult primary.^21^ It is hypothesised that a complex interplay between the tumour microenvironment, as well as immune, genetic and epigenetic features may make the primary lesion regress or remain dormant,^22^ although early invasive and metastatic behaviour is justifiably assumed, uncoupling proliferation from dissemination.

Finding a unifying molecular ‘CUP signature’ to explain the CUP phenotype remains elusive. Researchers have found considerable mutational heterogeneity between patient’s tumours. A handful of recent studies have elucidated common molecular pathways that are interrupted and may explain the aggressive and early dissemination pattern of CUP tumours. In 17 relapsed CUP tumours aberrations that affected cell cycle arrest, combined with either epigenetic deregulation or cell signalling activation, namely PI3K and MAPK signal transduction pathway alterations, were found consistently across the tumours analysed and the authors proposed a role for novel combinational therapies.^23^ A further study, employing genome wide transcriptome analysis, compared 41 CUP tumours with a molecularly predicted TOO to 186 metastatic lesions from 10 known tumour types. The authors describe CUP tumours as more distinct from their predicted primary tumour than the metastatic lesions from their confirmed primary tumour types. CUP tumour transcripts were enriched for DNA damage and repair genes, lacked tumour protein expression and showed more chromosomal instability, when compared to metastases of known tumour types.^24^ The authors suggest this supports the hypothesis of parallel progression in these cases but it may also be a reflection of the aggressive and advanced nature of the disease. Although these studies suggest some commonality in the biology of CUP they include small numbers of patients. Conversely, in 40 patients with favourable CUP, subtype microRNA signatures were compared to analogous known tumour type and showed no significant differences in expression, suggesting at least within the favourable subtype CUP tumours are biologically similar to their analogous primary.^25^

In the absence of a unique biology that unifies CUP tumours as a single entity, it is most likely that the CUP syndrome is a limitation of primary tumour determination. There remains an unexplained biology behind primary tumour regression, early dissemination, and aggressive metastatic phenotype. It is generally believed that CUP tumours, if a primary tumour of origin can be predicted, will behave and respond to treatments similarly to the predicted tumour type.^26^ Further molecular characterisation of larger numbers of CUP tumours would increase our understanding of the heterogeneity of the syndrome and determine if molecular CUP subtypes exist that may respond better to existing or novel treatments. Meanwhile, predicting TOO remains valuable to identify those patients that may benefit from alternative treatments.

## Tissue-of-origin (TOO) studies in CUP

Although debated, TOO classifiers are seen as a gateway to improving survival and therapeutic options in patients with CUP. Cancer treatment decisions are currently based on histological classification; without a TOO, treatment options are limited and access to newer therapies is difficult. There is an assumption that administering therapeutics based on the predicted primary tumour site will improve response rates and survival in patients with CUP.

Molecular methods applied to tumour tissue are able to predict the TOO, using gene expression profiling, gene microarrays, microRNA and DNA methylation analysis. Analysing known primaries generates a tumour-type gene signature database for classifying cancers, and this can predict the primary origin of known tumour types with accuracies of 80-95%.^27–29^ Many of these TOO classifiers have been successfully applied to CUP tumours to aid diagnosis (Table 1).^30–39^ However, applying these approaches in CUP tumours is limited by the nature of the disease; no primary tumour exists to validate these predictions. Instead, each TOO classifier is dependent on the known tumours that were selected to ‘train’ the classifier, with the predictions therefore limited by the range of tumour types used to generate the input; a veritable ‘catch 22’. Some classifiers have only used a small number of tumour types and their accuracy is not always reported.^38,40,41^ Some studies have attempted to validate predictions in CUP using autopsy data, latent primary emergence, or further immunohistochemistry stains.^36,39^ Gene expression profiling has been directly compared to IHC, within known metastatic tumour types, for accuracy in determining the primary tumour origin. Accuracy of gene expression profiling was 89%, compared with 83% for IHC when only one round of IHC determined the diagnosis. Gene expression profiling outperformed IHC (83% to 67%, respectively) in poorly differentiated cancers and those requiring a second round of IHC.^42^ This suggests that in the more difficult cases, as observed in CUP, gene expression profiling could be useful.

**Table 1:** Selected tissue-of-origin studies applied to CUP using molecular profiling of tumour tissue DNA (adapted from 1)

Study type; Lead author, year; Ref.	No. of patients profiled/ enrolled	Method analysis	No of tumour types	Prediction accuracy	Commonest predicted tumour types	Validation / Impact on clinical outcomes with prediction-based treatment
Retrospective; Horlings, 2008^30^	38	GEM; RNA	10	61%-93%	Lung (24%); CRC (18%); Pancreas (16%); Ovarian (11%)	Clinicopathological features and IHC / ND
Retrospective /prospective; Varadhachary, 2008^31^	104/120	10-gene qRT-PCR; RNA	6	61%	CRC (49%); NSCLC (33%); Pancreas (21%); Ovarian (14%)	Clinicopathological features / Improved OS of prospective vs retrospective cohort
Prospective; Varadhachary, 2011^32^	74/104	48-microRNA qRT-PCR	17	84%	CRC (*n* = 13); Ovarian (n = 6); SCC (*n* = 6); Pancreaticobiliary (*n* = 10); NSCLC (*n* = 6)	Clinicopathological features and IHC / ND
Retrospective; Fernandez, 2012^33^	42	DNA methylation array	19	78% (7/9 tumours)	CRC (34%); NSCLC (17%); Breast (17%)	Clinicopathological features / ND
Retrospective, Hainsworth, 2012^34^	42 predicted to be CRC	92-gene qRT-PCR, RNA	30	54-86%	Only studied CRC	Clinicopathological features / ND
Retrospective; Pentheroudakis 2013^35^	85/93	64 microRNA	42	92%	ND	Clinicopathological features and IHC / ND
Prospective; Hainsworth, 2013^36^	252/289	92-gene qRT-PCR; RNA	30	ND	Biliary tract (18%); CRC (10%); NSCLC (7%); Breast (5%); Urothelial (11%); Pancreatic (5%)	ND / Improved OS compared to historical controls
Retrospective; Tothill, 2015^37^	49	GEM; RNA	18	78%	SCC (*n* = 9); Lung (*n* = 7); kidney (*n* = 5); CRC (*n* = 4); cholangiocarcinoma (*n* = 2); mesothelioma (*n* = 2); Breast (*n* = 4)	Clinicopathological features / ND
Prospective phase II; Yoon, 2016^38^	38/46	2000-gene GEM; RNA	15	ND	NSCLC (21%); CRC (18%); Ovarian (18%); Pancreas (16%)	ND / Improved OS in platinum-responsive tumour types
Retrospective; Moran, 2016^39^	216	EPICUP DNA methylation array	38	87-100%	NSCLC (20%); Head and neck SCC. (10%); Breast (9%); CRC (9%); HCC (7%); Pancreatic (7%)	Latent primary, clinicopathological features, autopsy / Improved OS some patients (*n* = 31)

*CRC* colorectal cancer, *GEM* gene expression microarray, *HCC* hepatocellular carcinoma, *IHC* immunohistochemistry, *NSCLC* non-small cell lung cancer, *ND* not done, *OS* overall survival, *qRT-PCR* quantitative reverse transcription polymerase chain reaction

International or national guidelines do not currently recommend routinely predicting the TOO in CUP tumours.^4,43,44^ This is due to a lack of randomised, prospective data demonstrating improved clinical outcomes when treatments are chosen based on the predicted TOO. Only a handful of studies (outlined in Table 1) have investigated the clinical outcomes of treating patients with CUP based on gene expression predictions. Hainsworth et al (2013) successfully profiled 252 patients with CUP with a 92 gene classifier, and a total of 194 patients had therapy directed to the predicted primary tumour. Median survival of these patients was 12.5 months, which compared favourably to historic trial data (median survival 9-10 months). Those patients predicted to have more responsive tumour types had improved survival compared to those patients with less responsive tumour type predictions (median survival of 13.4 months compared to 7.6 months, respectively).^36^ Those patients with less responsive tumours had very poor prognosis, highlighting the heterogeneity of CUP tumours and the difficulties conducting CUP trials. Interestingly, retrospective analysis of those patients predicted to have NSCLC identified 4 patients with ALK rearrangements, one of which was treated successfully with second line crizotinib.^45^

A recent but small prospective phase II trial enrolled 46 patients with confirmed CUP treated with carboplatin, paclitaxel and everolimus. Thirty-eight of these patients were successfully molecularly profiled with a 2000-gene expression microarray. In 50% (*n* = 19) of cases the gene expression profiling predicted a tumour type that was likely to respond to platinum-doublet chemotherapy, including NSCLC, bladder, breast and ovarian cancer. The other half of the patients were predicted tumour types where empiric platinum-doublet chemotherapy would not be standard of care, including colon, pancreatic and hepatocellular carcinoma. Patients who were predicted to have platinum-responsive tumour types had significantly longer progression-free survival (PFS) and overall survival (OS), compared to those tumour types where platinum chemotherapy is not standard of care (PFS median 6.4 vs. 3.5 months, *P* = 0.026; HR 0.47 (95% CI: 0.24–0.93) and OS median 17.8 vs. 8.3 months, *P* = 0.0052; HR 0.37 (95% CI: 0.18–0.76)).^38^

Finally, a recent large but retrospective study predicted the TOO based on DNA methylation profiles of tumour tissue with 99·6% specificity (95% CI 99·5–99·7) and 97·7% sensitivity (96·1–99·2). Of 216 tumours assayed, 31 patients had therapy directed to the predicted TOO. An improved OS of 13.6 months was observed in these patients, compared to 6 months in the 61 patients who received empiric therapy (hazard ratio [HR] 3·24, *p* = 0·0051 [95% CI: 1·42–7·38]; log-rank *p* = 0·0029).^39^

These studies support the assumption that CUP patients treated with site-specific therapy could have improved clinical outcomes, and patients predicted to have tumours that are poorly responsive to known therapies may benefit from clinical trials. However, these studies are limited by a lack of randomisation, small numbers of patients treated and/or survival data compared to historical controls.

Importantly, these gene-expression profiling studies have aided the identification of a favourable colorectal-subtype of CUP. Two retrospective studies (Table 1) revealed that patients with CUP predicted to be a colorectal (CRC) primary performed significantly better when treated with colorectal chemotherapy regimens.^31,34^ With the emergence of improved chemotherapy regimens for CRC, survival of patients with a colorectal subtype of CUP is now similar to that of patients with metastatic CRC, where OS now approaches 24 months.^46,47^ Of note, within these studies, not all patients had a typical CRC IHC profile (CK20^+^/CK7^−^ or CDX2^+^), and without gene expression profiling they would not have been classified as CUP colorectal-subtype. ESMO CUP guidelines include both histopathologically and molecularly confirmed CRC within this favourable clinical subtype, despite not routinely recommending the molecular profiling of CUP tumours.^43^

The evidence base is currently not robust enough to recommend routine molecular TOO predictions for diagnosis in CUP. These studies do highlight a role for identifying subtypes of CUP that may gain clinical improvement with site-directed therapy, as demonstrated for the colorectal subtype of CUP. In addition, a significant proportion of patients with adenocarcinoma CUP are predicted to have lung cancer as their primary tumour type.^31,36,38,39^ Importantly, these patients could have EGFR and ALK fusion protein and PDL1 testing, where administration of appropriate targeted therapy significantly improves OS.^48–50^

## Using molecular features of CUP to predict therapeutic strategy

There is a movement away from traditional cancer classification by tissue and organ type alone, with more cancers now being sub-classified not only by histological subtype but also by their molecular characteristics.^51^ Increasingly, patients are treated with a precision oncology approach; therapy selection is based on molecular characteristics of the tumour and targeted to the biology of the disease process. As our molecular understanding of cancers improves, for example from comprehensive datasets such as TCGA, common molecular characteristics of cancer are discovered and new classifications are born.^52^ It is predicted 1 in 10 cancer patients might be classified (and possibly treated) differently using molecular classifications of cancer.^53^ Modern trial designs now reflect the known molecular heterogeneity within cancer types and so-called ‘basket trials’ include patients based on shared molecular drivers irrespective of their tumour type. Patients with CUP could gain huge benefits from such an approach and are the epitome of requiring personalised treatments. To date, no molecular sub-classification for CUP tumours exists and only a limited number of CUP tumours have been molecularly characterised to elucidate a role for targeted therapies.

### Targeted Therapeutics

The discovery of multiple driver mutations has paved the way for novel targeted therapeutics across multiple tumour types. Many of these molecular drivers are not tumour-type specific and are common across several tumour types. Therapies targeting these driver mutations have had a significant impact on response rates and survival in previously poor-prognosis metastatic tumours.

Several groups have investigated the mutational landscape of CUP tumours to elucidate driver mutations and potential roles for targeted therapy (Table 2).^23,54–59^ Significant mutational heterogeneity exists, and mutations found in CUP are found amongst many different cancer types. For example, KRAS mutations are found in 30-50% of colorectal cancers and up to 25% of lung cancers.^60–62^ Studies investigating the presence of driver mutations and molecular aberrations in CUP vary widely in their conclusions regarding whether changes are ‘potentially druggable’ (ranging from 15-96%). Only one phase I trial to date has reported early clinical follow-up data of 11 patients with CUP that have been matched to therapies based on molecular aberrations; four of those patients treated had stable disease and one a mixed response.^23^ Within the literature there are numerous case reports of patients with CUP being treated successfully with targeted therapies (Supplementary Table S1)^45,63–68^; however, the inherent bias of case studies needs to be considered, including the fact that they are non-randomised observational studies and often with selective reporting and publication of positive findings.

**Table 2:** Summary of genetic aberrations found within CUP tumours from tissue biopsy

Year; Ref.	Technique	No. of patients	% of samples with at least one genomic aberration	% potentially therapeutically targetable mutation	Most common genetic aberrations
2015;^23^	Retrospective DNA sequencing 236 genes and 47 introns from FFPE (based on FoundationOne assay)	200	96%	20%	*TP53* (55%)
					*KRAS* (20%)
					*CDKN2A* (19%)
					*MYC* (12%)
2014;^49^	Retrospective gene mutation analysis (47 genes) and protein expression from tumour tissue	1806	ND	96%^a^	*TP53* (38%)
					*KRAS* (18%)
					*BRCA2* (11%)
					*PIK3CA* (9%)
					*STK11* (6%)
2013;^50^	Retrospective DNA sequencing (701 genes) and CNA of tumour tissue	16	100%	81%	*TP53* (62%)
					*NOTCH1* (18%)
					*CDKN2A* (18%)
2014;^51^	CTNNB1, MET, PIK3CA, KRAS, BRAF mutation targeted sequencing from FFPE	87	66%	36.9%^b^	*CTNNB1* (19.5%)
					*KRAS* (10.2%)
					*PIK3CA* (6.7%)
					*MET* (4.5%)
					*BRAF* (4.5%)
2016;^52^	Retrospective 50 targeted genes and copy number analysis	55	84%	15%	*TP53* (55%)
					*CDKN2A* (22%)
					*KRAS* (18%)
					*SMAD4* (11%)
					*EGFR* (1%)
2017;^53^	Next generation sequencing of tumour tissue	17	88%	41%	Impaired *P* signalling (47%)
					Epigenetic deregulation (47%)
					Impaired cell cycle control (47%)
2018;^54^	Retrospective 592-gene NextSeq platform panel	389	ND	22%	*TP53* (54%)
					*KRAS* (22%)
					*ARID1A* (13%)
					*PIK3CA* (9%)
					*CDKN2A* (8%)
					*SMARCA4* (7%)

*ARID1A* AT-Rich interaction domain 1 A, *BRCA2* breast cancer 2 gene, *CDKN2A* cyclin-dependent kinase Inhibitor 2A, *CAN* copy number analysis, *CTNNB1* catenin beta-1, *EGFR* epidermal growth factor receptor, *FFPE* fresh frozen paraffin embedded, *KRAS* Kirsten rat sarcoma Viral Oncogene Homolog, *ND* not documented, *PIK3CA* p100α catalytic subunit 1A phosphatidylinositol 3-kinase, *pts*  patients; *ref*. reference, *STK11* serine/threonine Kinase 11, *TP53* tumour protein 53

^a^Based on mutations and protein expression profiles indicated therapeutic benefit of targeted agents, cytotoxics and immunotherapy

^b^Proportion of patients with activating mutations

The prognostic or predictive significance of driver mutations are tumour-type specific and therefore their value in CUP is uncertain without a TOO. For example, it is well documented that BRAF mutations are predictive of response to BRAF inhibitor monotherapy in melanoma, but not in colorectal carcinoma.^69^ A recently completed basket trial assessing the efficacy of the BRAF inhibitor Vemurafenib in multiple BRAF mutant solid tumours confirmed a variable response across different tumour types. Response rates were highest in NSCLC (42%) but poor in CRC (0%) and cholangiocarcinoma (12%), despite the majority of cases having the same V600E somatic BRAF mutation. These results exemplify the challenges of predicting response to targeted therapy based on single driver mutations alone.^70^ A recent study evaluating genetic aberrations in CUP highlighted that the common pathways interrupted may require combinatorial therapies for clinical responses, and this should be considered when designing trials for targeted drugs.^23^

### Immunotherapy

Immunotherapies are emerging as important therapeutics in several cancer types, with promising response rates and in some cases long-term durable responses in the metastatic setting. It would be reasonable to expect that a subset of patients with CUP may respond to immunotherapy. Markers of responsiveness to immunotherapy are under intense research and debate. Those currently under investigation in known tumour types include immune biomarker expression, high tumour mutational burden (TMB), and microsatellite instability (MSI) and mismatch repair deficiency (dMMR).

There are two case reports describing responses to immunotherapy in patients with CUP; one patient with high expression levels of programmed cell death ligand-1 (PDL1), and the other had confirmed dMMR (Supplementary Table S1).^66,68^ Recently published works have demonstrated the efficacy of pembrolizumab in dMMR and MSI-high tumours irrespective of tumour cell origin;^71,72^ consequently, pembrolizumab is the first drug to gain pan-cancer FDA approval for use in any MSI-high/dMMR solid tumour. The frequency of high TMB, PDL1 expression and MSI-high amongst CUP tumours has been evaluated by a few studies, which suggest one or more of these biomarkers may be present in up to 28% of cases.^55,59,73^ Determining a potential immunotherapy-responsive subgroup of CUP is hampered by a lack of validated predictive biomarkers and further research is desperately needed. The opening of the Roche Foundation Medicine Phase II global CUP trial (NCT03498521) will make some progress in evaluating the efficacy of novel molecularly-guided therapies, including immunotherapy, in patients with unfavourable CUP and may aid biomarker discovery.

## Research limitations within CUP studies

Evaluating CUP research is hampered by the lack of international consensus on the definition and classifications of CUP. Furthermore, trials investigating CUP treatment strategies are limited by the diagnostic heterogeneity. Many of the predicted tumour types in CUP TOO studies will respond to the standard of care chemotherapy regimens recommended for the unfavourable CUP subtype (platinum-based doublet chemotherapy). This makes direct comparisons of this treatment with site-specific chemotherapy difficult, due to significant treatment overlap.

A tumour biopsy, usually of the primary tumour, is currently the ‘gold standard’ for tumour evaluation and diagnosis, and thus determination of initial treatment. Unfortunately there are inherent limitations in both acquisition and interpretation of tumour biopsies, together with uncertainty over whether a single tumour biopsy, which is often very small, may be representative of the entire tumour landscape.^74^ There is a significant degree of both inter- and intra-tumour heterogeneity within known tumour types, as well as clonal and sub-clonal variations between metastatic sites in the same patient.^75^ Tissue biopsies taken from single metastatic sites are less likely to determine the tumour origin by IHC than biopsies taken from the primary tumour,^76^ and are also unlikely to capture the heterogeneity of the cancer burden, which has direct implications for treatment response and resistance. Tumour biopsies are invasive, sometimes anatomically difficult to obtain and the procedure is not without risk of harm to the patient; they are therefore usually performed only once - at the point of diagnosis. However, our emerging understanding of treatment resistance mechanisms, especially with targeted agents, has meant it is increasingly important for repeated evaluation of the tumour at progression to better understand tumour evolution and guide appropriate therapy.

## The Role of the Liquid Biopsy and CUP

**Fig. 2:**
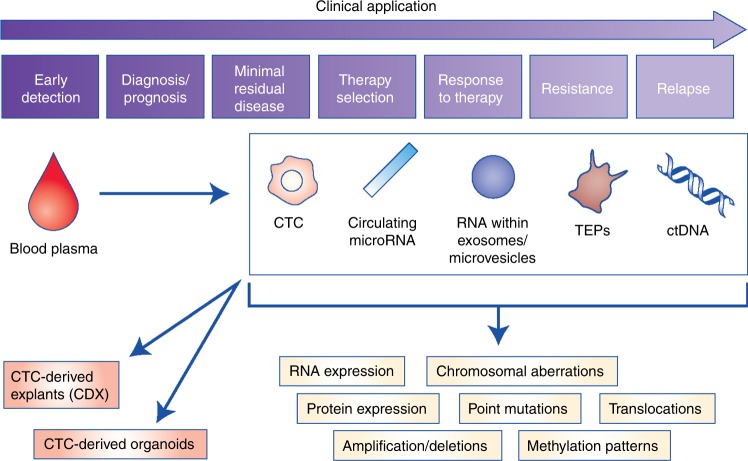
Potential clinical and research applications of liquid biopsies for the management of CUP. Clinical and research applications of liquid biopsies are wide-reaching, from early detection and diagnosis to monitoring response to therapy and earlier detection of disease relapse. Liquid biopsies contain genetic information from the tumour in the form of circulating tumour cells (CTCs), tumour-educated platelets (TEPs), mircoRNAs contained within exomes and circulating free tumour DNA (ctDNA). ctDNA is a component of circulating free DNA (cfDNA); fragments of DNA either passively released by cells as a consequence of apoptosis and cell death, or actively released by cells as a potential messaging signal.^111^ Patients with cancer have a much higher proportion of cfDNA in the blood compared with healthy normal volunteers (HNV); a greater proportion is tumour-derived ctDNA that is shed from the highly proliferating tumour cells and/or tumour cell death.^112,113^ CTCs are released into the bloodstream by a passive process of tumour shedding or through active intravasation, including processes such as epithelial-to-mesenchymal transition (EMT), cell-to-cell cooperation and vasculogenic mimicry.^91,92^ Epithelial cells undergoing EMT lose their characteristic cell-to-cell interactions, become mobile and gain invasive properties. CTCs that underwent EMT may reverse this process during the process of metastasis formation; however, only a very small proportion of CTCs subsequently propagate a distant metastasis.^74^ Molecular analyses that can be performed on genetic material include copy number alterations, actionable mutation detection, amplifications, and deletions, as well as epigenetic and transcriptome analysis. CTCs can be implanted into immune-compromised mice as CTC explants (CDX) or cultured directly as CTC organoids for drug testing

Since the discovery of tumour-derived genetic material within the blood stream, the concept of a blood sample, or ‘liquid biopsy’, to identify clinically useful tumour biomarkers has gained research interest.^77^ Liquid biopsies (Fig. 2) have the potential to overcome many of the limitations of a tumour biopsy; notably being less invasive, meaning acquisition of serial samples is easier and more acceptable to the patient. Liquid biopsies are a rapidly evolving field within cancer and hold the potential to improve screening, diagnosis, treatment stratification and therapy monitoring. Here we explore the potential role for liquid biopsy in CUP including blood-based TOO classifiers, the feasibility of liquid biopsies in CUP and how further research may enable its clinical utility.

Blood-derived cancer proteins, for example CA125 and PSA, have been used for many years as surrogate biomarkers for disease. Unfortunately, these markers lack the specificity to be used as a screening tool and have limited sensitivity, in some cases, for their use in diagnosis and assessment of treatment response or relapse.^78^ Improvements in nucleic acid sequencing technologies has enabled the detection of low quantities of tumour genetic material within the blood and show the potential to be both sensitive and specific to an individual’s tumour.^79^ These blood-based biomarkers include circulating tumour DNA (ctDNA), tumour microRNAs (miRNAs) and platelet-derived tumour mRNA, as well as analysis of DNA, RNAs and protein expression from individual circulating tumour cells (CTCs) (Fig. 2).^77,80^

### cfDNA and ctDNA

A wealth of recent advances in liquid biopsies have shown them to be clinically useful for tumour biomarker detection in known tumour types; for example, blood-based EGFR mutation testing in NSCLC is used within the clinic to detect circulating tumour DNA (ctDNA) harbouring common EGFR mutations that predict response/resistance to EGFR inhibitors.^81^ There are, however, very few studies investigating the role of liquid biopsies in CUP (summarised in Table 3). ctDNA is a useful liquid biomarker because its molecular characteristics reflect that of the tumour and are a ‘real-time’ reflection of the tumour’s physiological processes. Several studies have investigated the role of cfDNA as a biomarker across numerous cancer types.^82^ There is evidence cfDNA can be used for diagnosis and early detection, prediction and prognostication post-surgery, in the metastatic setting and as a surrogate for tumour burden, monitoring of response to treatment, and as an indicator of relapse and resistance (reviewed in ref. ^83^).

**Table 3:** Summary of liquid biopsy research in CUP and blood-based Tissue-of-Origin studies

Lead Author; Year; ref.	Liquid biopsy approach	Patients included	Study results
*Liquid biopsies in cancer of unknown primary*			
Allard; 2004^94^	CTC enumeration (CellSearch®)	964 cancer patients (11 CUP); 244 non-malignant/healthy individuals	CTCs detected in 52% of CUP samples (n = 27); mean CTC count 16 ( + /-35). The second highest proportion of positive samples amongst tumour types.
Komine; 2014^96^	CTC enumeration (CellSearch®)	10 patients with CUP (5 treatment naive)	CTCs detected in 50% of samples. CTC counts 3-207 (median = 31). CTC count declined with treatment in one patient
Pentheroudakis; 2012^97^	CTC (IF detection)	24 patients with CUP	CTCs detected in 15/24 (62.5%) patients but of no prognostic value
Kato; 2017^68^	cfDNA mutational profile	442 patients with CUP	Targeted NGS (up to 70 genes) detected mutations in 66% of patients. The most common alterations were: *TP53* (37.1%); *KRAS* (18.6%); *PIK3CA* (15.4%); *BRAF* (7.5%); *MYC* (7.5%). cfDNA mutations altered during treatment and aided therapy decisions in one patient.
*Blood-based tissue-of origin studies*			
Best; 2015^80^	TEPs	55 healthy donors; Tumour types: 60 NSCLC; 41 CRC; 39 glioblastoma; 35 pancreatic cancer; 39 breast cancer; 14 HPB cancer	mRNA profiles of TEPs able to predict tissue of origin from 6 primary tumour types by support vector machine classifier with median accuracy of 73%
Klein; 2018^103^	cfDNA	749 controls; 878 cancer cases:28 CRC; 19 oesophageal; 5 Head and neck; 5 HPB; 73 lung; 17 lymphoma; 11 MM; 10 ovarian; 10 pancreatic	Targeted NGS (507 genes), copy number variation, whole genome bisulfite sequencing. Sensitivity 60-90% in detecting cancer in those tumour types (stages I-III)
Cohen; 2018^104^	cfDNA	626 cancer cases (ovarian, lung, liver, stomach, pancreatic, breast, CRC, oesophageal); 812 healthy donors	16 gene and 8 tumour protein panel (CancerSEEK) identified the cancer type by supervised machine learning in a median of 69-98% of patients
Sun; 2015^105^	cfDNA	29 HCC patients; 32 control subjects	Plasma DNA tissue mapping from cfDNA methylation patterns determined liver tissue contribution was higher in HCC patients compared to controls.
Lehmann-Werman; 2016^106^	cfDNA	42 patients with pancreatic cancer; 47 healthy subjects	cfDNA methylation patterns of pancreatic cell death identified in 20/42 patients with pancreatic cancer. Performed better than cfDNA *KRAS* mutation detection
Guo; 2017^107^	cfDNA	75 normal individuals; 29 lung cancer; 30 CRC	cfDNA methylation patterns predicted tissue of origin in 82.8% of CRC samples and 88.5% of lung cancer patients
Matthew; 2016^108^	CTCs enrichment (CellSearch®)	2 patients with breast cancer; 1 patient with prostate cancer	IHC staining of isolated CTCs able to determine tissue of origin in breast and prostate cancer using CK7, CK20, TTf-1, ER, PSA stains.
Lu; 2016^109^	CTC enrichment (CMx chip)	12 healthy individuals; 13 patients with cancer (lung, CRC and prostate)	Distiguished cancer from healthy individuals and determined tissue of origin by IHC staining of CK7, CK20, TF-1, CDX2 and PSA.

*cfDNA* circulating-free DNA, *CDX2* caudal Type Homeobox 2, *CK* cytokeratin, *CRC* colorectal carcinoma, *CTC* circulating tumour cells, *CUP* cancer of unknown primary, *HCC* hepatocellular carcinoma, *HPB* hepatobiliary, *IF* immunofluorescence, *KRAS* Kirsten rat sarcoma viral oncogene homolog, *MM* multiple myeloma, *NGS* next-generation sequencing, *NSCLC* non-small cell lung cancer, *PIK3C* p100α catalytic subunit 1 A phosphatidylinositol 3-kinase, *PSA* prostate specific antigen, *ref*. reference, *TEP* tumour-educated platelet, *TP53* tumour protein 53, *TTF1* thyroid transcription factor 1

To date, only one group has used patient cfDNA to profile the mutational landscape of CUP tumours. Evaluating 442 patients with targeted sequencing of 54-70 genes, they found 80% of patients harboured at least one genetic alteration. The mutational profile was comparable to studies profiling CUP tumour tissue, and demonstrated heterogeneity similar to that seen across all tumour types. In one patient, serial on-treatment ctDNA samples demonstrated dynamic changes in the mutation profile that were consistent with response and then resistance to therapy.^68^ This indicates the feasibility of using ctDNA to not only profile tumour but also to track resistance mechanisms whilst on therapy. In known tumour types we know that ctDNA can be used to detect specific cancer mutations and track response and resistance to therapies and predict relapse prior to radiological progression.^84,85^ For patients with CUP, there is a clinical need for early non-invasive indicators of treatment response or resistance, such that futile treatments can be halted and detrimental side effects can be avoided.

One limitation of cfDNA mutational analysis is determining the biological significance and pathogenicity of mutation results, given that mutations can be found in non-malignant/healthy individuals.^86^ It is therefore apparent that mutational profiling alone can only stratify small numbers of patients to possible therapeutic targets, and only a proportion of these patients will gain benefit from these treatments. Epigenetic alterations, such as DNA methylation, histone modification and microRNA-mediated gene regulation, assert transcriptional control and regulate gene expression without genetic modification. Emerging evidence suggests these alterations may provide a more useful analysis of DNA and overcome the uncertainty of mutational analysis, as they better reflect gene expression and therefore function. It is thought that epigenetic modifications play a key role in tumour initiation and cancer predisposition, and epigenetic changes are tumour and tissue-type specific.^87^ Epigenetic biomarkers are already in use in the clinical setting and potentially useful in predicting TOO from CUP tumour tissue.^39^ Several groups have demonstrated that epigenetic changes in ctDNA can be a useful biomarker for cancer prognosis, prediction of treatment response and post-operative detection of minimal residual disease (reviewed in ref. ^88^).

### Circulating tumour Cells (CTCs)

CTCs are primary tumour or metastatic cells that have entered the peripheral circulation as singular cells or as cell clusters that are termed circulating tumour microemboli (CTM).^74^ Notably, the majority of CTCs are undergoing some stage of apoptosis in the bloodstream, which contributes to ctDNA levels.^89,90^ The exact mechanisms underlying haematogenous dissemination of CTCs from the primary and/or metastatic tumour are not fully understood, but this may be a passive process of tumour shedding or through active intravasation, including epithelial-to-mesenchymal transition (EMT), cell-to-cell cooperation and vasculogenic mimicry.^91,92^ Epithelial cells undergoing EMT lose their characteristic cell-to-cell interactions, become mobile and gain invasive properties,^93^ and CTCs that have undergone EMT may reverse this process during the process of metastasis formation, though only a very small proportion of CTCs subsequently propagate a distant metastasis.^74^

CTCs are rarely found in healthy normal volunteers or in patients with benign disease,^94^ but they are found in many cancer types. The number of CTCs present varies between cancer type and patient, and their observed prevalence also depends on the technology platform used for their detection. CTCs are promising as a liquid biopsy and various clinical studies have confirmed the negative prognostic significance of CTCs on survival in numerous tumour types, most notably in breast, colorectal and prostate cancer (reviewed in^95^).

Patients with CUP often have a high tumour burden and aggressive clinical course. It would be reasonable to expect that these patients may have a relatively high frequency of CTCs, reflecting their metastatic capability, as well as high ctDNA levels, reflecting rapid tumour cell proliferation and death. A handful of studies have evaluated CTCs in a small number of patients with CUP (Table 3).^68,94,96,97^ The most recent publication evaluated 10 CUP patients and found CTCs in 50% of cases (median CTC count: 31). CTCs were more frequent in treatment-naive patients and in one patient CTC levels decreased with treatment.^96^ Two of the studies used the CellSearch® platform; this is the only FDA-approved CTC technology that detects EpCam-positive CTCs. It therefore fails to detect CTCs that have down-regulated this epithelial marker, for example during invasion whilst undergoing epithelial-to-mesenchymal transition. New marker-independent approaches, such as the RareCyte and HDSCA which were developed on the principle that ‘no cell is left behind’, should improve CTC assay sensitivity because all nucleated cells in the sample are captured onto slides, allowing staining with a flexible panel of markers and physical picking of single cells for molecular characterisation.^98^ Application of these approaches to CUP should improve the recovery and detection of CUP CTCs that may have lost their epithelial markers by nature of the disease.

Isolation of CTCs has the further advantage of allowing functional analysis of the single tumour cell, including whole-genome sequencing, gene expression profiling, epigenetic studies, and proteomics. In vivo models from cultured CTCs have been established from patients with advanced cancer (lung and melanoma) and exhibiting high CTC counts.^99,100^ Small-cell lung cancer CTC-derived explants faithfully reflected tumour responses to chemotherapy, indicating that these may be ideal models for understanding tumour resistance mechanisms.^101^ Therefore, these CTC-derived explant (CDX) models may allow for novel drug testing and discovery,^99^ and may serve as an informative way to investigate tumour heterogeneity. If successfully applied to CUP, this could reveal insights into the biology and heterogeneity of CUP tumours and allow for rational drug testing to improve therapeutic options for patients with CUP. Of note, a recent publication described a similar approach using tumour tissue-derived cell culture and patient-derived explants (PDX) from a patient with neuroendocrine CUP. Successful pathway analysis and drug trial analysis in these models identified AKT pathway activation and response to an AKT inhibitor in the absence of actionable mutations.^102^

### Determining the TOO from a liquid biopsy

A single blood test that can detect and diagnose the cancer type is seen as the ‘holy grail’ of cancer diagnostics, and rapid advancements in the field of liquid biopsies is bringing this closer to reality. Numerous recent proof-of-concept studies in common tumour types claim sensitive and specific cancer detection with liquid biopsies (Table 3). Methods include genetic and epigenetic profiling of cfDNA, staining of CTCs, and mRNA analysis of tumour-educated platelets (TEPs).^80,103–109^ Many of these studies have focussed on early detection of disease in search of a non-invasive cancer screening tool. However, we know that ctDNA mutation detection is more sensitive in advanced tumours,^110^ reflecting higher tumour burden and increased circulating genetic material from the tumour. The TOO studies report sensitivities of 60-98% dependent on tumour type and molecular approach.^80,103–107^ These studies demonstrate that liquid biopsy molecular characterisation is feasible, especially in the metastatic setting. Predicting the TOO via molecular profiling is a debated topic within CUP; these methods have yet to be applied to CUP tumours and will likely be more challenging. CUP tumours are by definition tumours without histological definition and therefore more difficult to molecularly classify. In addition, multiple metastatic deposits commonly observed in patients with CUP are likely to increase ctDNA heterogeneity. CUP tumours may have lost obvious ‘molecular signposts’ to the TOO, which may make CTC phenotyping unlikely to be helpful. Although research in this area is very exciting, utility of any of these methods remains to be seen with regards to determining the TOO in patients with CUP.

**Fig. 3:**
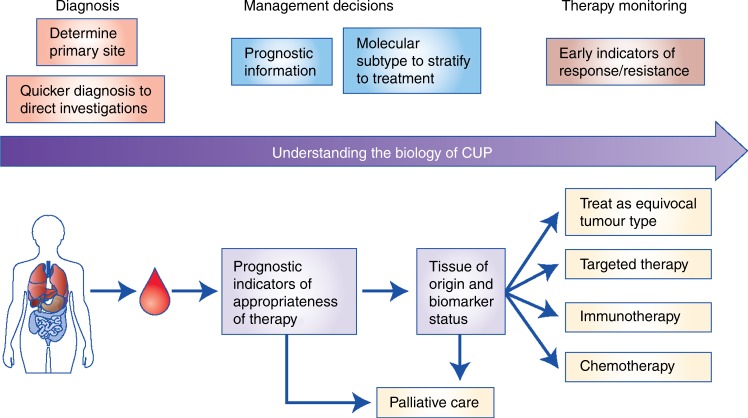
Proposed role of liquid biopsies in CUP research and future diagnosis, management stratification and monitoring of CUP patients. A single blood test at presentation of CUP could determine tissue of origin, evaluate prognostic and predictive biomarkers and stratify patients to appropriate treatment. Serial blood samples could monitor early response to therapy or resistance, enabling timely switch or halting of futile treatments. EGFR epidermal growth factor receptor, IHC immunohistochemistry, MSI microsatellite instability, PS performance status.

The limited studies evaluating ctDNA and CTCs in CUP demonstrate its feasibility, but only with further research will we establish if it is clinically useful and applicable. TOO classifiers using liquid biopsies show exciting potential for diagnosis within known tumour types but have yet to be applied to patients with CUP. It may be invaluable in streamlining the diagnostic pathway for patients with CUP and MUO, as well as the rapid diagnosis of potentially curable cancers where diagnosis is time critical, for example germ cell carcinoma and lymphoma. The most useful liquid biopsies will most likely be multi-analyte; not only predicting TOO, but also providing better prognostication, detecting actionable mutations and markers of response to chemotherapy and/or immunotherapy, and enabling early response or resistance monitoring (Fig. 3).

## Summary and the future of CUP

CUP remains an unmet clinical and research need. A lack of validated prognostic and predictive biomarkers means the scientific rationale for existing and novel therapy selection is lacking, with therapeutics in clinical development remaining out of reach. A small proportion of patients within the favourable subset respond well to treatments of their analogous tumour type, and achieve meaningful improved survival. However, the majority of patients comprise the unfavourable subtype, and are currently treated with combination chemotherapy based on clinical and radiological characteristics and limited histopathological information. This ‘one-size-fits-all’ approach does not reflect the heterogeneity of these tumours, and emphasises the need for better treatment stratification.

Within CUP, as well as other tumour types, there remains an important need to find novel biomarkers, ideally liquid-based, that better predict response to chemotherapy, immunotherapy and targeted therapy. There is also a need for robust evidence before implementation of liquid biopsies in the clinical management of CUP, and it is therefore necessary for well-designed and adequately powered clinical trials to prospectively incorporate liquid biomarker discovery into the trial design (Supplementary Box 1). Patients with a good performance status should especially be offered enrolment in well-designed clinical trials. Liquid biopsies would allow the study of CUP heterogeneity in more detail and at a molecular level, whilst providing a less invasive and longitudinal means of monitoring disease. Ultimately, we need validated prognostic and predictive biomarkers to stratify patients appropriately and to inform on better therapeutic options. This approach will hopefully lead to improved clinical outcomes for CUP patients, but also has the potential to improve our understanding of metastatic cancer as a whole (Fig. 3).

## Electronic supplementary material

Supplementary Table S1 - Summary of published case studies of patients with CUP treated with targeted and novel therapies

Supplementary Box 1
