# Supplementary material for: Molecular characterisation and liquid biomarkers in Carcinoma of Unknown Primary (CUP): taking the ‘U’ out of ‘CUP’
Source: Br J Cancer. 2018 Dec 23;120(2):141–53. doi: 10.1038/s41416-018-0332-2 (PMC6342985; doi:10.1038/s41416-018-0332-2)
Supplement: Supplementary file 2 — Supplementary Box 1 [file 41416_2018_332_MOESM2_ESM.docx]

**Supplementary Box 1: Translation of liquid biopsies into modern trial design**

- It is clear a shift within oncology to a precision medicine approach can dramatically improve therapeutic options and survival in metastatic cancer. Recent advances with targeted and immunotherapy hold the promise of longer term disease control in metastatic cancer of known tumour types and it is hopeful that these improvements can be translated to CUP.
- The heterogeneity of the CUP syndrome along with its dismal prognosis and limited treatment options make patients with CUP prime candidates for a personalised medicine approach. We would argue many patients would benefit from entry into well designed clinical trials with translational research at the forefront to better understand CUP biology.
- Newer modern trial design including basket and umbrella trials provide opportunities to test multiple treatments on the patients most likely to benefit from them. Traditionally, patients with CUP were often excluded from clinical trials but there are an increasing number of basket trials recruiting irrespective of tissue of origin.
- However, it is vitally important that we have accurate predictive and prognostic biomarkers to stratify patients to the right treatments and monitor disease reliably and sensitively to detect early resistance and stop expensive and futile treatments at the appropriate time. Incorporating liquid biomarker discovery into trials will help this.
